# THE INSECT ACHETA DOMESTICUS IS A PROMISING MODEL FOR AGING AND AGING INTERVENTION STUDIES

**DOI:** 10.1093/geroni/igae098.2443

**Published:** 2024-12-31

**Authors:** Gerald Liao, Jackson Wezeman, Warren Ladiges

**Affiliations:** University of Washington, Seattle, Washington, United States; University of Washington, Seattle, Washington, United States; University of Washington, University of Washington, Washington, United States

## Abstract

A number of animal species are being used as models to study aging and age-related diseases. They have varying advantages such as shorter lifespan, relatively low costs, and full access to internal organs. While no single model can replicate all aspects of human aging, there are unexplored non-mammalian species that may have additional advantages. The domestic house cricket (Acheta domesticus) has a short lifespan, a simple but well-defined organ system, an omnivorous diet from young to old, very low maintenance costs, and neurogenesis in a hippocampus-like brain structure. A preliminary study investigated effects of flax oil, rich in omega-3 fatty acids, on lifespan in adult crickets. Cold pressed flax oil was added to moistened guinea pig chow mash as a 10% mixture and fed to crickets starting at 8 weeks of age and continuing for 11 weeks when the last cricket died. Results demonstrated a significant extension in survival for crickets fed the flax oil diet, emphasizing the low cost, simplicity, and short time required for dietary studies in this species. This observation provides the rationale for additional studies to characterize the house cricket as a promising model for aging and aging intervention investigations.

